# Evaluation of drug susceptibility profile of *Mycobacterium tuberculosis* Lineage 1 from Brazil based on whole genome sequencing and phenotypic methods

**DOI:** 10.1590/0074-02760200520

**Published:** 2021-01-29

**Authors:** Arthur Emil dos Santos Guimarães, Abhinav Sharma, Ismari Perini Furlaneto, Liliana Rutaihwa, Jedson Ferreira Cardoso, Marília Lima da Conceição, Lizânia Borges Spinassé, Edson Machado, Maria Luiza Lopes, Rafael Silva Duarte, Sebastien Gagneux, Philip Noel Suffys, Karla Valéria Batista Lima, Emilyn Costa Conceição

**Affiliations:** 1 Universidade do Estado do Pará, Instituto de Ciências Biológicas e da Saúde, Pós-Graduação em Biologia Parasitária na Amazônia, Belém, PA, Brasil; 2 Instituto Evandro Chagas, Seção de Bacteriologia e Micologia, Ananindeua, PA, Brasil; 3 International Institute of Information Technology, Department of Data Science, Bangalore, India; 4 University of Basel, Basel, Switzerland; 5 Swiss Tropical & Public Health Institute, Basel, Switzerland; 6 Centro de Inovações Tecnológicas, Instituto Evandro Chagas, Ananindeua, PA, Brasil; 7 Universidade Federal do Espírito Santo, Núcleo de Doenças Infecciosas, Vitória, ES, Brasil; 8 Fundação Oswaldo Cruz-Fiocruz, Instituto Oswaldo Cruz, Laboratório de Genética Molecular de Microrganismos, Rio de Janeiro, RJ, Brasil; 9 Universidade Federal do Rio de Janeiro, Instituto de Microbiologia Professor Paulo de Góes, Laboratório de Micobactérias, Rio de Janeiro, RJ, Brasil; 10 Fundação Oswaldo Cruz-Fiocruz, Instituto Oswaldo Cruz, Laboratório de Biologia Molecular Aplicada a Micobactérias, Rio de Janeiro, RJ, Brasil; 11 Fundação Oswaldo Cruz-Fiocruz, Instituto Nacional de Infectologia Evandro Chagas, Programa de Pós-Graduação em Pesquisa Clínica e Doenças Infecciosas, Rio de Janeiro, RJ, Brasil; 12 Fundação Oswaldo Cruz-Fiocruz, Instituto Nacional de Infectologia Evandro Chagas, Laboratório de Bacteriologia e Bioensaios em Micobactérias, Rio de Janeiro, RJ, Brasil

**Keywords:** tuberculosis, genomic, drug resistance, Mycobacterium tuberculosis, Lineage 1, Brazil

## Abstract

**BACKGROUND:**

The evaluation of procedures for drug susceptibility prediction of *Mycobacterium tuberculosis* based on genomic data against the conventional reference method test based on culture is realistic considering the scenario of growing number of tools proposals based on whole-genome sequences (WGS).

**OBJECTIVES:**

This study aimed to evaluate drug susceptibility testing (DST) outcome based on WGS tools and the phenotypic methods performed on isolates of *M. tuberculosis* Lineage 1 from the state of Pará, Brazil, generally associated with low levels of drug resistance.

**METHODOLOGY:**

Culture based DST was performed using the Proportion Method in Löwenstein-Jensen medium on 71 isolates that had been submitted to WGS. We analysed the seven main genome sequence-based tools for resistance and lineage prediction applied to *M. tuberculosis* and for comparison evaluation we have used the Kappa concordance test.

**FINDINGS:**

When comparing the WGS-based tools against the DST, we observed the highest level of agreement using TB-profiler. Among the tools, TB-profiler, KvarQ and Mykrobe were those which identified the largest number of TB-MDR cases. Comparing the four most sensitive tools regarding resistance prediction, agreement was observed for 43 genomes.

**MAIN CONCLUSIONS:**

Drug resistance profiling using next-generation sequencing offers rapid assessment of resistance-associated mutations, therefore facilitating rapid access to effective treatment.

Tuberculosis (TB) is a millenary infectious disease caused by bacteria belonging to the classical taxonomy group *Mycobacterium tuberculosis* complex (MTBC). Within this group, *M. tuberculosis* and *M. africanum* are the main causative agents of the disease in humans which has been classified into eight phylogenetic lineages (L1-L8), presenting different patterns of geographical distribution and associated drug resistance profile. Among these, L1 is mostly restricted to Eastern Africa and the South of India.^1^

Lineage 1 is infrequently reported in South America except for one study that described 98 such strains in Northern Brazil, representing 10% of the study population.^2^ Through single nucleotide polymorphism (SNP) typing, we hypothesised to have been introduced during the trans-Atlantic slave trade.^3^

The L1 has not been associated to the drug resistance (DR),^4^ different to L2 and L4 that are strongly associated with DR.^4^ We therefore determined the DR profile using the conventional phenotypical antimicrobial drug susceptibility tests (DST) and compared that with *in silico* DR prediction on whole-genome sequencing (WGS) of 71 isolates of *M. tuberculosis* L1 from Pará generated presently, together with three isolates with recently published genomes.^5^ *In silico* procedures were: TB-profiler,^6^ KvarQ,^7^ PhyresSe,^8^ Mykrobe,^9^ MTBSEQ,^10^ CASTB^11^ and Resistance Sniffer (RSniffer).^12^

## MATERIALS AND METHODS

*Sampling* - Out of 980 *M. tuberculosis* isolates from the state of Pará, Brazil, 97 were classified as East-African-Indian (EAI) by Spoligotyping,^2^ among which 71 were recovered and classified as Lineage 1^3^ were used in this present study for DR evaluation.

*Drug susceptibility testing* - DST for isoniazid (INH), rifampicin (RIF), ethambutol (EMB) and streptomycin (SM) was performed using the Proportions Method in Löwenstein-Jensen (LJ) medium using the recommended critical concentrations and using the H37Rv strain as a control. The DST was performed according to the national manual for laboratory surveillance of TB and other mycobacteria^13^ without modifications. This test consisted of detecting the proportion of resistant bacilli present in a sample of *M. tuberculosis*, given the concentration of the drug, capable of inhibiting the development of sensitive cells, but not that of resistant cells - “critical concentration”.

*Whole-genome sequencing* - After DNA extraction by Phenol-chloroform protocol^5^ and library preparation using the Nextera XT DNA Library Prep Kit (Illumina, San Diego, USA), the isolates were sequenced using the Hiseq 2500 platform (Illumina, San Diego, USA) with a coverage of 250x. The raw reads were deposited at NCBI under the accession number PRJNA494931^5^ and PRJNA630228.

Following the genome quality control by FastQC v0.11.9, reads were trimmed using Trimmomatic v0.35.^14^ To compare the drug susceptibility profile based on SNPs obtained from WGS, we have used the following tools on the trimmed files: TB-profiler v2.8.6,^6^ KvarQ v0.12.2,^7^ PhyresSe,^8^ Mykrobe v0.8.1,^9^ MTBSEQ v1.0.4,^10^ CASTB^11^ and RSniffer.^12^ All results are described in the Supplementary data **(Table I)**.

*Statistics* - To compare the sensitivity, specificity and accuracy of DST as determined phenotypically or *in silico*, the Kappa Concordance Analysis was applied using the BioEstat 5.5 software.^15^ This test is a measure of interobserver agreement and measures the degree of agreement beyond what would be expected only by chance. This measure of agreement has a maximum value of 1, where this value 1 represents total agreement and values close to and even below 0 indicate no agreement, or the agreement was exactly what was expected by chance. An eventual Kappa value less than zero or negative, suggests that the agreement found was lower than that expected by chance. Therefore, it indicates disagreement, but its value has no interpretation as a degree of disagreement. The p-value is considered significant when it is less than or equal to 5% (p ≤ 0.05).

*Ethics* - This study was approved by the Ethics Committee/IEC, Ananindeua, Pará, Brazil, under the number 018/2011 (CAAE: 0002.0.071.000-11).

## RESULTS

Based on the DST, among the 71 isolates, 38 (53.5%) were drug susceptible, 17 (23.9%) were resistant to at least one of the drugs and 16 (22.5%) were TB-MDR. The summary of the results obtained with each of the WGS based tools for TB resistance prediction is described in Table I.

**TABLE I:** Drug resistance profile according to whole-genome sequencing tools for first-line anti-tuberculosis

Genomic tools	Multidrug resistant	Other resistance	Susceptible
TB-profiler	18 (25.4%)	14 (19.7%)	39 (54.9%)
PhyResSe^*a*^	1 (1.4%)	25 (36.2%)	43 (62.4%)
KvarQ	18 (25.4%)	13 (18.3%)	40 (56.3%)
CASTB^*b*^	15 (25%)	34 (56.7%)	11 (18.3%)
RSniffer	0	0	71 (100%)
Mykrobe	18 (25.4%)	17 (23.9%)	36 (50.7%)
MTBSEQ	17 (23.9%)	23 (32.4%)	31 (43.7%)

*a*: n = 69; *b*: n = 60.

Among the tools TB-profiler, KvarQ and Mykrobe identified the largest number of TB-MDR cases, while PhyResSe presented a low capacity to find mutations related to the *rpoB* gene (k = 0,08). Due to technical issues by not generating data with the PhyResSe and CASTB softwares, we were unable to obtain results for all submitted genomes, reducing the total number of samples to 69 and 60, respectively. All samples submitted to RSniffer were determined as being drug susceptible.

When comparing the WGS based tools to DST (Table II), we observed the highest level of agreement on all drugs in the case of TB-profiler (Table III). The program that showed the least compatibility^16^ with all antibiotics was RSniffer. The conclusion for each tool is described in Supplementary data **(Table II)**.

**TABLE II:** Comparison among the seven whole-genome sequencing based tools against the drug susceptibility test

Drug-susceptibility test (Proportion Method) →		INH		RIF		PZA		EMB		SM			MDR: RIF+INH	
WGS tools ↓	DST status	R	S	R	S	R	S	R	S	R	S	MDR-status	MDR	N-MDR
TB-profiler	R	26	1	17	5	3	2	8	6	6	1	MDR	14	4
	S	4	40	1	48	3	63	0	57	2	62	N-MDR	1	52
MTBSEQ	R	28	10	15	5	2	5	7	5	6	2	MDR	13	4
	S	2	31	3	48	4	60	1	58	2	61	N-MDR	3	51
Phyresse^*a*^	R	20	0	1	0	2	5	8	4	5	2	MDR	1	0
	S	10	41	17	53	4	60	0	59	3	61	N-MDR	15	55
Kvarq	R	26	1	17	5	1	4	7	2	6	1	MDR	14	4
	S	4	40	1	48	5	61	1	61	2	62	N-MDR	2	51
CASTB^*b*^	R	25	3	13	4	2	4	5	5	5	36	MDR	11	4
	S	5	38	5	49	2	61	3	58	3	27	N-MDR	5	51
Resistance sniffer	R	0	0	0	0	0	0	0	0	0	0	MDR	0	0
	S	30	41	18	53	6	65	8	63	8	63	N-MDR	16	55
Mykrobe	R	27	2	17	6	4	5	7	7	6	1	MDR	14	4
	S	3	39	1	47	2	60	1	56	2	62	N-MDR	2	51

INH: isoniazid; RIF: rifampicin; PZA: pyrazinamide; EMB: ethambutol; SM: streptomycin; MDR: multidrug-resistant; WGS: whole-genome sequencing; DST: drug susceptibility test. *a*: n = 69; *b*: n = 60.

**TABLE III:** TB-profiler *versus* MTBSEQ whole-genome sequencing tools according to Kappa Coefficient

	Kappa (Interpretation)		Observed concordance		Replicability		95% CI		p-value	
Drugs	TB-profiler	MTBSEQ	TB-profiler	MTBSEQ	TB-profiler	MTBSEQ	TB-profiler	MTBSEQ	TB-profiler	MTBSEQ
INH	0.854	0.692	92.96%	84.51%	Almost perfect	Substantial	0.731 to 0.977	0.528 to 0.856	<0.0001	<0.0001
RIF	0.792	0.713	91.55%	88.73%	Substantial	Substantial	0.635 to 0.949	0.528 to 0.898	<0.0001	<0.0001
PZA	0.508	0.238	92.96%	87.32%	Moderate	Fair	0.133 to 0.882	-0.108 to 0.585	<0.0001	<0,0219
EMB	0.645	0.653	90.41%	91.55%	Substantial	Substantial	0.411 to 0.879	0.400 to 0.906	<0.0001	<0.0001
SM	0.776	0.718	95.77%	94.73%	Substantial	Substantial	0.533 to 1.000	0.457 to 0.980	<0.0001	<0.0001

INH: isoniazid; RIF: rifampicin; PZA: pyrazinamide; EMB: ethambutol; SM: streptomycin; 95% CI: 95% confidence interval.

Considering the performance of the *in silico* DST against the antibiotics separately, Mykrobe was the one with highest accuracy in relation to INH (k = 0.855 and p < 0.0001). Owing to the design of algorithm or technical runtime issues, CASTB presented a greater number of positive results for SM, influencing the agreement (k = 0.04 and p < 0.2025) together with EMB (k = 0.166 and p < 0.095), these results do not indicate statistical significance. For pyrazinamide (PZA), *in silico* analysis demonstrated a low agreement rate, with Kappa coefficient results ranging from 0.114 to 0.508 [Supplementary data **(Table III)**].

Among the evaluated tools in general, TB-profiler performed favorably. For identification of MDR samples however, sensitivity (77%), specificity (96%) and accuracy (14.71) were the same for KvarQ and Mykrobe [Supplementary data **(Table III)**].

A Venn graphic illustrates the comparison among the four most sensible tools (TB-profiler; KvarQ; Mykrobe and MTBSEQ) and conventional DST, including 43 genomes as common elements: 12 MDR (G04875, G04876, G04877, G04878, G04882, G04893, G049162, G049222; G049392; G049442, G049512 and G049522), 26 susceptible (G04871, G04881, G04883, G04885, G04886, G04887, G04889, G04896, G049182, G049202, G049212, G049252, G049272, G049292, G049302, G049312, G049372, G049402, G049412, G049422, G049432, G049462, G049472, G049492, G049532 and G049542) and five INH monoresistant isolates (G04888, G049382, G049482, G049502 and G049582) (Figure).

**Figure:**
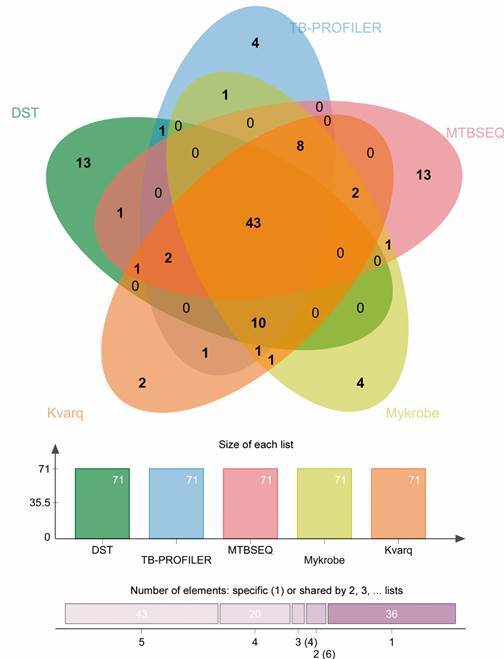
Comparison among five drug resistance prediction tools based on whole-genome sequencing data against the drug resistance testing (DST) reference technique for 71 *Mycobacterium tuberculosis* Lineage 1 from Brazil.

## DISCUSSION

One of the objectives of the Genomic Era is to replace the classic genotyping techniques for the detection and identification of MTBC species for diagnostic purposes and the phenotypic methods for DST, by *in silico* analysis of WGS data.^17^ During the last decades, genotyping tools have been developed that identify both lineage and drug resistance and their validation is of major importance to evaluate their impact as a possible substitute for traditional methodologies.

This present study compared the widely used WGS based tools to predict antimicrobial resistance profile in 71 genomes from isolates of *M. tuberculosis* of the Lineage 1 from Pará, Brazil, using DST as the reference method. The DST based on the proportions method is mostly used in Brazil as an AST for mycobacteria, but it is a laborious and time-consuming method, requiring four to six weeks to obtain the results.^18^ On the other hand, DR prediction from WGS data can be performed from early positive MGIT cultures after an average of 14 days, or even directly on sputum sample generating results within five days.^19^

Lineage 1 (EAI) is not usually associated with DR and has also low correlation to transmissibility and virulence, presenting a restricted geographical distribution.^4^ ^,^ ^20^ In this study however, the resistance profile by the DST demonstrated that 17 (23.9%) were resistant at least to one drug and 16 (22.5%) were MDR. This high frequency of MDR isolates might be related to the fact that the TB cases were from the reference hospital for MDR-TB Hospital Universitário João de Barros Barreto (HUJBB), including TB contacts (without a previous history of TB), and patients suspected of treatment failure or TB relapse. Compared the DR of Lineage 1 in the context of other lineages from the same region, the most predominant was Lineage 4, among of which T and X lineages, were associated to MDR-TB, while Lineage 1 the highest among ‘any resistance’ group.^2^

Among all *in silico* based tools tested presently, we encountered difficulties to predict resistance to PZA, which can be partly due to alternative mechanisms of resistance to this drug (non *pnc*A related)^21^ and reports of low-frequency SNPs that may be associated with PZA resistance.^22^ In the present study however, we did not include PZA in the conventional DST, a major limitation of the study.

The ability to correctly identify whether there is a mutation in the sample is called sensitivity and the ability to identify whether the sample does not actually have the mutation is specificity, when analysing these results it is important to generate the level of accuracy, thus it is easier to assess whether the results obtained were compared correctly.

TB-profiler showed that, in addition to good sensitivity and specificity,^23^ it has a good statistical correlation with conventional DST, proving that it is a good resistance predictor tool. A recent study on isolates from patient from the state of São Paulo in Brazil and from province of Sofala in Mozambique compared DST performed in liquid medium MGIT-960 SIRE kit against TB-profiler prediction and the LPA tests Genotype-MTBDRplus 2.0 and MTBDRsl 2.0. The TB-profiler had the best performance among the genotypic DST as compared to the phenotypic test with a good concordance with phenotypic DST for RIF and SM (89.6%), INH (96.5%) and EMB (82.7%). WGS sensitivity and specificity for detection resistance were respectively 87.5 and 92.3% for RIF; 95.6 and 100% for INH; 85.7 and 93.3% for SM while 100 and 77.2% for EMB.^24^

Moreover, our data is also in agreement with other studies^10^ ^,^ ^25^ suggesting that the use of TB-profiler together with Mykrobre, MTBSeq and KvarQ may increase the chances to fully elucidate the mutations of the genomes under analysis.

Regarding RIF prediction by PhyResSe, it detected only one mutation (rpoB_His445Arg) in *rpoB* gene of a MDR strain by DST (G04893), therefore this tool presented a low sensitivity and specificity for this drug. In general, we observed a lower performance PhyResSe compared to other pipelines, much more pronounced that that described in other studies.^23^ This might be a characteristic of the performance of this pipeline particular in genomes of Lineage 1 and needs further investigation.

Even though in some studies, CASTB has demonstrated a good performance in finding variants related to mutations,^11^ ^,^ ^21^ we observed a high number of false positives for resistance to SM, in addition to not generating outputs for some samples resulting in inconclusive results.

Regarding RSniffer based genome analysis, all isolates were considered as susceptible to all drugs and this seems to be due to the fact that by default, this tool assumes Linage 1 as a drug pan-susceptible,^12^ limiting its applicability in this strain population.

Since WGS is mainly done from a DNA pool of a culture, it is possible that there is a discrepancy between the phenotypic and genotypic tests for the same sample, as these strains can manifest themselves, thus tests can be influenced by mixed infections or mixtures of drug susceptible and resistant populations in phenomenon of heteroresistance.^26^

The evaluated tools in this study were based on the technique of Direct Association (DA) which relies on the established correlation between the various resistance conferring mutations and their presence or absence in the MTB isolate under study. These pre-documented correlations are utilised by these tools to ascertain the drug resistance profile of the sample.^27^ ^,^ ^28^ Studies which are valid as genomic analysis protocol for the detection of MTBC species and their genetic characterisation, especially for resistance analysis, are important for the progress of translational research in TB, with the goal to replace phenotypic tests by WGS.

Recently, WGS performed directly on clinical specimen has been proposed for an even more rapid TB surveillance, allowing researchers to do real-time genomic epidemiology and drug resistance surveillance in settings where culture and DST are not available.^29^ ^,^ ^30^ However, this is still technically challenging and a under active study.
